# A Versatile Porous Silver-Coordinated Material for the Heterogeneous Catalysis of Chemical Conversion with Propargylic Alcohols and CO_2_

**DOI:** 10.3390/nano9111566

**Published:** 2019-11-05

**Authors:** Lu Yang, Yong Dou, Zhen Zhou, Daopeng Zhang, Suna Wang

**Affiliations:** 1 School of Chemistry and Chemical Engineering, Shandong University of Technology, Zibo 255000, China; yanglu@sdut.edu.cn (L.Y.);; 2 State Key Laboratory of Fine Chemicals, Dalian University of Technology, Dalian 116024, China; 3 Shandong Provincial Key Laboratory of Chemical Energy Storage and Novel Cell Technology, School of Chemistry and Chemical Engineering, Liaocheng University, Liaocheng 252059, China; wangsuna@lcu.edu.cn

**Keywords:** metal-organic framework, heterogeneous catalysis, carbon dioxide fixation

## Abstract

The efficient transformation of carbon dioxide into useful chemical feedstock is of great significance, attracting intense research interest. The widely studied porous-coordinated polymers possess large pores to adsorb guest molecules and further allow the contact and to transfer the substrate molecule within their microenvironment. Here we present the synthesis of a silver-based metal-organic frameworks (MOFs) material with a three-dimensional structure by incorporating a tetraphenyl-ethylene moiety as the four-point connected node via the solvothermal method. This polymer exhibits as an efficient heterogeneous catalyst for the carboxylative cyclization of CO_2_ to α-methylene cyclic carbonates in excellent yields. Moreover, the introduction of silver (Ag (I)) chains in this framework shows the specific alkynophilicity to activate C≡C bonds in propargylic alcohols to greatly accelerate the efficient conversion, and the large pores in the catalyst exhibit a size-selective catalytic performance.

## 1. Introduction

Recently the utilization and transformation of carbon dioxide as a C1 source into valuable chemical products has become important for research purposes and attractive for both chemists and environmentalists [1,2,3]. Until now, a great deal of effort has been made to develop porous materials towards the adsorption and fixation of carbon dioxide [4,5,6,7,8]. Within the numerous examples, the porous coordination polymers, the metal-organic frameworks (MOFs), have been regarded as an excellent platform because of their intriguing structural diversity, modular nature and facile tenability [9,10,11,12]. Moreover, in terms of catalysis, these materials usually possess rich open metal sites, which are capable of CO_2_ activation and further converting into catalysis products, highlighting the advantages of MOF materials for applications in many fields [13,14,15,16].

Silver (Ag (I)) complexes, which function as σ- and/or π-Lewis acids, have been shown to be powerful catalysts for alkyne transformations due to their d^10^ electronic configurations, favoring interactions with the carbon–carbon π-bond of the alkynes, referred to as alkynophilicity [17,18]. The superior alkynophilicity leads to the facilitating of the π-coordination of Ag∙∙∙C≡C bonds by nucleophilic attack. 

Therefore, the silver (I) ion is considered as one of the most ideal activators of the carbon–carbon triple bond in many usable organic reactions, including cycloaddition, hydroazidation and cross-coupling reactions starting with alkynes and its derivatives [19,20]. Among them, the carboxylative cyclization of propargylic alcohols and carbon dioxide to α-methylene cyclic carbonates, which are important natural products with potential bioactivity and chemical value, have shown a great promise pathway for the direct incorporation of carbon dioxide, and have achieved considerable reactivity and selectivity catalyzed by many kinds of Ag-based catalysts via the benign π-activation [21,22].

**Figure 1:**
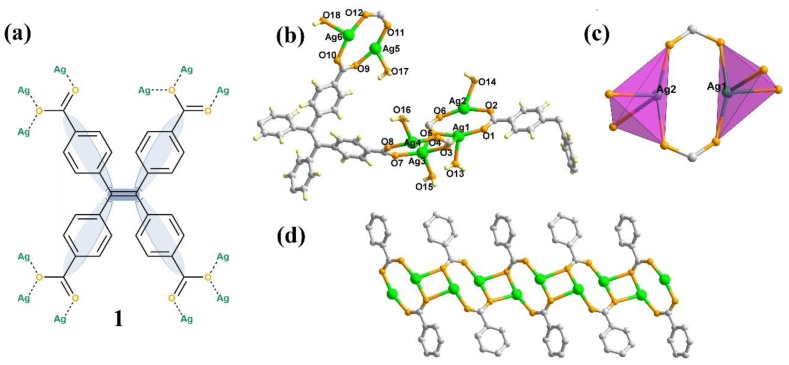
(**a**) The representation of ligand tetrakiscarboxyphenylethylene (TCPE) and the coordinated modes with Ag ions; (**b**) Ball-and-stick representation of **1** in an asymmetric unit with atomic-numbering scheme; (**c**) The coordination environment of Ag1 and Ag2 with tetrahedral fashion; (**d**) Connected mode of Ag chains. Color code: Ag, green; O, orange; C, gray. H atoms are omitted for clarity.

Inspired by these considerations, we envision that the combination of the alkynophilic Ag (I) ion into the porous and unique structural features of MOFs makes it possible to develop a potential synthetic strategy to construct efficient heterogeneous catalysts for the reactions starting with alkynes and its derivatives [23,24,25]. Herein, we present the synthesis of silver-based MOFs materials, (noted as **1**, Figure 1a), with a three-dimensional structure by the incorporation of the tetrakis(4-carboxyphenyl)ethylene (H_4_TCPE) moiety as the four-point connected node via the solvothermal method. The catalytic properties for the carboxylative cyclization of carbon dioxide were investigated by performing the reaction starting with propargylic alcohols and carbon dioxide.

## 2. Materials and Methods

### 2.1. General Experimental Details

All chemicals were of reagent grade quality obtained from commercial sources and used without further purification. All epoxides were purchased from Acros (Shanghai, China) and distilled under a nitrogen atmosphere from calcium hydride (CaH_2_) prior to use. Carbon dioxide (CO_2_, 99.995%) was purchased from the Dalian Institute of Special Gases (Dalian, China), and was then used as received. The elemental analyses of C, H and N were performed on a Vario EL III elemental analyzer (Frankfurt, Germany). Inductively-coupled plasma (ICP) analysis was performed on a Jarrel-AshJ-A1100 spectrometer (Waltham, MA, USA). The powder XRD diffractograms were obtained on a Rigaku D/MAX-2400 X-ray diffractometer (Tokyo, Japan) with a Cu sealed tube (λ = 1.54178 Å). IR spectra were recorded as KBr pellets on a NEXUS instrument (Madison, WI, USA). Thermogravimetric analyses (TGA) were carried out at a ramp rate of 10 °C min^−1^ in nitrogen flow with a SDTQ600 instrument (TA Instruments, New Castle, DE, USA). Gas sorption isotherms were measured using an Autosorb-IQ-C analyzer of Quantachrome (Boynton Beach, FL, USA). The CO_2_ adsorption isotherms for desolvated compounds were collected in a relative pressure range from 10 to 1.0 × 10^5^ Pa. Liquid UV-Vis spectra were performed on a TU-1900 spectrophotometer (Beijing, China). ^1^H spectra were recorded on a Varian INOVA-400 MHz type spectrometer (Varian, Palo Alto, CA, USA). Their peak frequencies were referenced versus an internal standard (TMS) shifts at 0 ppm for ^1^H NMR.

### 2.2. Preparation of **1**

All reagents were used as purchased without further purification. Tetrakis (4-carboxyphenyl) ethylene (H_4_TCPE) was prepared according to the literature methods [26] and characterized by ^1^H NMR.

Synthesis of 1: A mixture of H_4_TCPE (30 mg, 0.06 mmol) and silver nitrate (AgNO_3_) (196.9 mg, 0.52 mmol) were dissolved into 4 mL dimethylformamide (DMF) in a screw-capped vial. The resulting mixture was kept in an oven at 100 °C for three days. After cooling the autoclave to room temperature, green block single crystals were separated, washed with water and air-dried. Yield: 10% (based on the crystal dried in vacuum). Anal. calcd. for C_90_H_54_Ag_12_O_27_ [Ag_12_(TCPE)_3_(H_2_O)_3_]: C 37.73, H 1.88, Ag 45.23%. Found: C 37.95, H 2.02, Ag 45.12%. IR (KBr): 3421 (br, s), 1677 (w), 1552 (w), 1532 (w), 1414 (s), 1196 (s), 1128 (s), 854 (w) and 763 (w) cm^−1^.

### 2.3. Crystallography

X-ray intensity data were measured on a Bruker SMART APEX CCD-based diffractometer (Mo–Kα radiation, λ = 0.71073 Å, Karlsruhe, Germany) using the SMART and SAINT programs [27,28]. The crystal data was solved by direct methods and further refined by full-matrix least-squares refinements on *F*^2^ using the SHELXL-97 software (Göttingen, Germany) and an absorption correction was performed using the SADABS program (Göttingen, Germany) [29,30]. The remaining atoms were found from successive full-matrix least-squares refinements on *F*^2^ and Fourier syntheses. Non-H atoms were refined with anisotropic displacement parameters. The hydrogen atoms within the ligand backbones were fixed geometrically at calculated distances and allowed to ride on the parent non-hydrogen atoms. Details of the crystal parameters, data collection and refinement are summarized in Table 1.

### 2.4. Catalysis Experiments

In a typical reaction, the catalytic reaction was conducted by adding propargylic alcohols (3 mmol), catalyst (0.3 mmol% loading, based on each Ag site), triphenylphosphine (Ph_3_P, 2.5 mmol%) and acetonitrile (CH_3_CN) (1 mL) in a 25 mL autoclave reactor, purged with 0.5 MPa CO_2_ under a constant pressure for 10 min to allow the system to equilibrate. The vessel was set in an oil bath with frequent stirring at the temperature of 50 °C for 30 h. At the end of the reaction, the reactor was placed into an ice bath for 20 min and then opened. The catalysts were separated by centrifugation, and a small aliquot of the supernatant reaction mixture was taken to be analyzed by ^1^H NMR to calculate the yields of the reaction. The organic phase was collected and then purified by flash column chromatography on silica gel using petroleum ether–ethyl acetate as an eluent to give the desired products.

## 3. Results and Discussion

### 3.1. Structure Description

The synthesis of **1** was starting with the raw materials of AgNO_3_ and H_4_TCPE under the solvothermal reaction at 100 °C with the yield of 10%. Elemental and powder X-ray diffraction (XRD) analyses indicated that the bulk samples of **1** consisted of a pure, single phase (Figure S5). Single-crystal X-ray structural analysis revealed that **1** crystallizes in *C2c* space group. There are six independent Ag ions and one and a half ligands in its asymmetric unit (Figure 1b). All the Ag (I) ions are four-coordinated in a tetrahedral fashion and possess the same coordination environment, which includes two *µ*_2_-O bridges, one *µ*-O atom and onelattice water molecules. The Ag (I) ions are connected to form an infinite 1D chains (Figure 1d).

**Figure 2:**
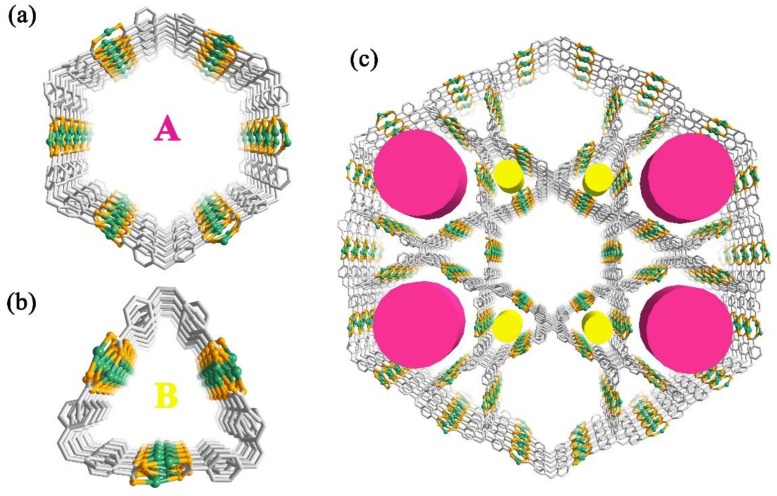
The triangular channel (**a**) and a hexagonal channel (**b**) in **1**; (**c**) Structure of **1** showing the three-dimensional network with one-dimensional channels.

The H_4_TCPE ligands are well-ordered arranged and the eight carboxylic groups in each ligand are all coordinated, acting as a *µ*_4_-bridge to connect each Ag chains extending the structure to the 3D frameworks (Figure 2, see Figures S1 and S2 in the supplementary materials for details). **1** possesses two kinds of channels along the *c* axis; including a triangular channel (A) and a hexagonal channel (B). These two channels are edge-sharing each other, with the overall lengths of the edges approximately 16.8 Å, and thus one hexagonal channel is surrounded by six triangular channels. The diagonal distances of the hexagonal channel are ranged from 31.1 to 33.8 Å. In the absence of guest molecules, the effective free volume of **1** calculated by PLATON program, was proven to be 59.3% of the crystal volume. The Ag chains are regarded as four-point-connected nodes, and the ligands are considered to be the linkers. The 3D framework of **1** can be depicted with the Schläfli symbol of 4^6^6^4^∙7∙8 (Figure S3). Notably, the type of this framework is similar to the reported MOF PCN-222, which self-assembly is by Zr ions and porphyrin ligand [31]. The major difference between these two MOFs is the vertex of each hexagonal and triangular one-dimensional open channel. Unlike the metal oxide Zr_6_ clusters as the vertices in PCN-222, the Ag chains in **1** are regarded as the edges of each hexagonal and triangular channel, and the C=C bonds of the TCPE ligands are located at the vertices (Figure S4).

### 3.2. XPS Analysis and CO_2_ Sorption Studies

**Figure 3:**
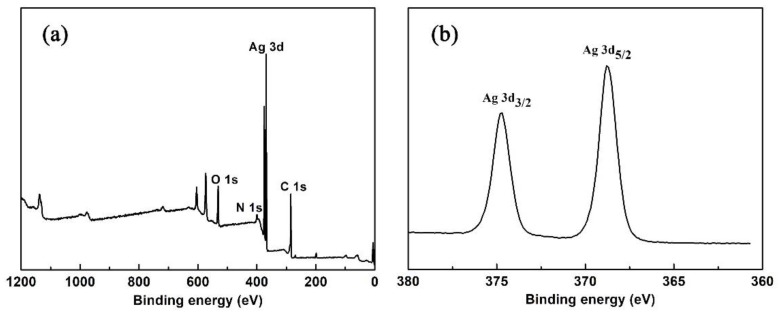
(**a**) XPS survey spectrum of **1**; (**b**) XPS spectrum of **1** for Ag 3d_3/2_ and Ag 3d_5/2_.

The XPS analysis of **1** was examined in the solid state. Both survey and high-resolution spectra of Ag 3d are shown in Figure 3. The binding energy for the C 1s peak at 284.8 eV was used as a reference. From the XPS survey spectrum, we can observe that the elements Ag, O and N were contained in **1**. In Figure 3b, two peaks at 374.7 and 368.8 eV are ascribed to Ag^+^ (3d_3/2_) and Ag^+^ (3d_5/2_), respectively [32].

To evaluate the porosity and affinity toward CO_2_ of this material, the CO_2_ sorption capability of **1** was evaluated. Before the measurement, the crystal samples were immersed in ethanol to exchange the uncoordinated solvent molecules. The PXRD pattern of the activated sample is a match with the fresh sample and calculated patterns, indicating the unchanged structure after desolvation (Figure S5), and the TG curve of **1** has also been presented (Figure S6). Then, the outgassing process was conducted at 100 °C under high vacuum for 6 h to obtain the activated samples. First of all, for further confirmation of the porosity, the N_2_ adsorption measurement of the activated **1** was carried out at 77 K (Figure S7) and this indicated a high uptake of 927.4 cm^3^ g^−1^. The Brunauer-Emmett-Teller (BET) surface area was calculated to be 1241.0 m^2^ g^−1^. 

**Figure 4:**
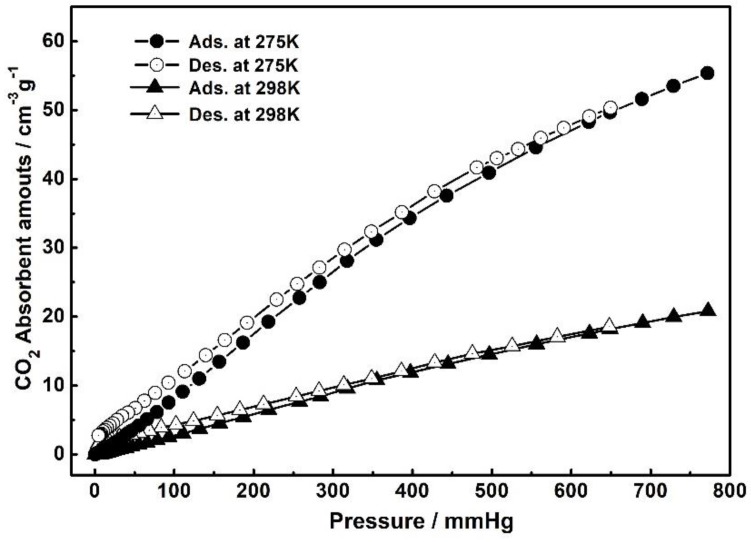
Gas sorption isotherms of **1** of CO_2_ measured at 275 and 298 K, respectively. Filled shape, adsorption; open shape, desorption.

As shown in Figure 4, The CO_2_ adsorption measurements for the activated **1** at 275 and 298 K are reversible, and the CO_2_ absorbent amounts show a steady rise tendency as the increase of pressure with the uptake of 55.4 cm^3^ g^−1^ (corresponding 2.47 mmol g^−1^) and 20.8 cm^3^ g^−1^ (0.928 mmol g^−1^) at 1 bar, respectively. These results give a strong evidence of the high porosity for this MOF material and the possibility of adsorbing the CO_2_ molecules within the pores. We envision that the high CO_2_ uptake ability could play a significant role in facilitating this material to activate and catalyze CO_2_ molecules as a C1 source in the following catalysis studies.

### 3.3. Dye Adsorption Properties

**Figure 5:**
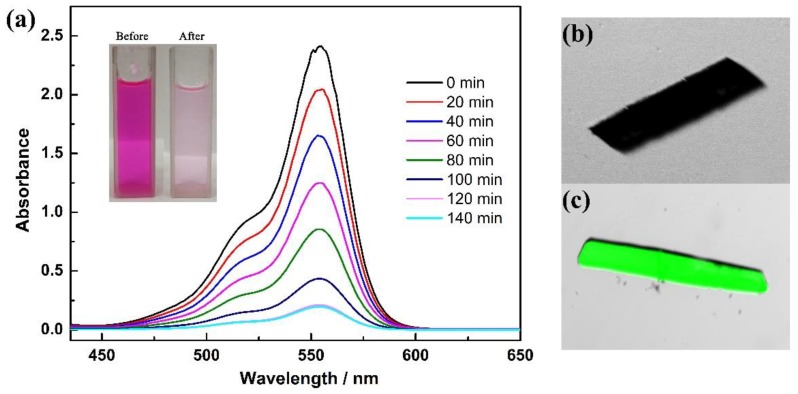
(**a**) UV-vis spectra of RhB after immersing **1** at different time intervals. Insert: The pictures of RhB solution before and after adsorption by **1**; Confocal images of empty (**b**) and soaked (**c**) RhB dye molecule of **1**.

To verify the ability of this porous material to accommodate guest molecules, the dye adsorption studies of **1** have been examined. The dye molecule rhodamine B (RhB, where the structure of RhB is shown in the supplementary materials as Figure S8) was chosen for the adsorption experiments, not only due to its excellent photophysical properties, such as long adsorption, long emission wavelength and high fluorescence quantum yield [33], but also the suitable kinetic size with the window scale of the hexagonal channel in **1**. By immersing the 5 mg sample **1** in 10 mL aqueous solution containing RhB, we can observe that the color of the solution has been gradually faded within 2 h. 10 µL supernatant of the solution was taken at several time intervals and diluted for UV-Vis analysis. As shown in Figure 5, the UV-Vis absorption spectra of RhB solution exhibited an intense absorption band centered at 554 nm, which is assignable to the typical absorption band of RhB. With the addition of **1**, the absorption intensity of the maximum absorption peak decreases from 2.4 to 0.2 in 120 min and no additional decrease was observed after that. These results indicate the absorption of RhB molecules, which are accessible into the pores of **1**. We speculate that the π⋅⋅⋅π interactions and potential hydrogen bonds between the framework and dye molecules play an intrinsic role in the adsorption process [34]. Additionally, the dye-adsorbed crystals, after the UV-vis measurement and being isolated from the solution, have been further examined by confocal laser scanning microscopy. The bright-field images and confocal images of the obtained samples were scanned at λ_em_ = 510–610 nm, exited by λ_em_ = 488 nm through a 405/488 nm filter. A strong green fluorescence response can be observed in the bright-field image that can be assigned to adsorbed RhB molecules. These results are consist with the UV-vis experiments and suggest that the accessible pores and adsorption ability of **1** provides the superiority for further catalysis studies.

### 3.4. Catalysis Studies

Our catalytic experiments were initially investigated for the carboxylic cyclization of propargylic alcohols as the model reacted with CO_2_. In a typical experiment, the reactions were conducted in an autoclave reactor using the propargyl alcohol (3 mmol) with CO_2_ purged to 0.5 MPa in 1 mL CH_3_CN at 50 °C. ^1^H NMR analysis reveal that catalyst **1** (0.3 mol% loading, based on each Ag site) could act as an effective heterogeneous catalyst for this catalysis reaction in the presence of 2.5 mol% triphenyl phosphine (Ph_3_P), which afforded the 97% yield, and the TON value is 320 within 30 h (Table 2). Control experiments suggest that no detectable conversion occurs in the absence of catalysts or the additive Ph_3_P, and we replaced the Ph_3_P with other additives (such as, TBUP, DBU and TEA), which also could not afford the high yields (20%, 32% and 18%, respectively). However, Ph_3_P itself was ineffective to this reaction under the given conditions.

**Figure 6:**
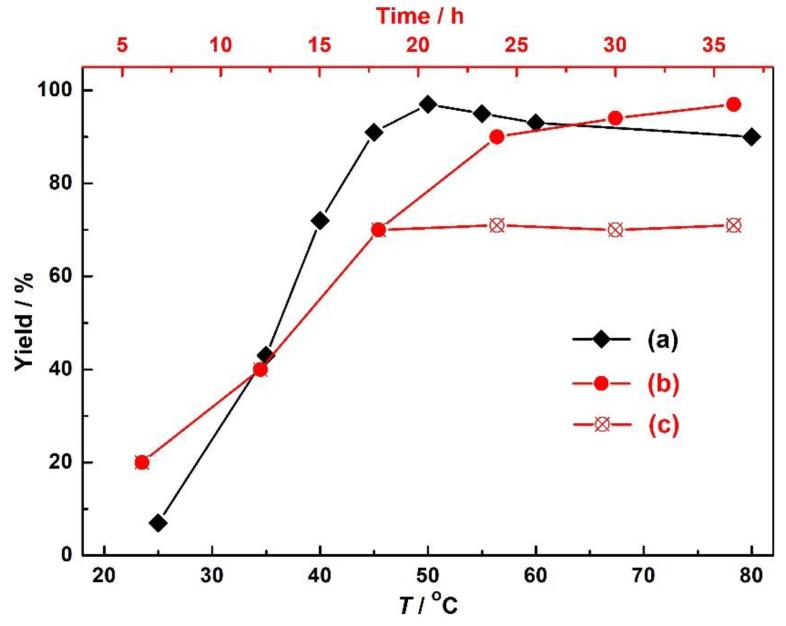
(**a**) The influences of the reaction temperature on the carboxylic cyclization of propargylic alcohols with CO_2_; (**b**) catalytic traces of the catalysis performed under optimal conditions and with the catalyst **1** filtered after 18 h (**c**).

The high yield of our Ag-based catalytic system was mainly attributed to the abound presence of Ag (I) ions capable of activating carbon–carbon triple bonds of the substrate propargyl alcohol. As for the catalyst **1**, this react ion was also carried out and underwent the smooth CO_2_ balloon at room temperature to afford a low yield of 4.9%, suggesting that relatively high temperature and slight CO_2_ pressure were required for the activation of the substrates. However, when we just increased the pressure of CO_2_ to 1 MPa, the reaction yields were not remarkably increased under other fixed conditions, and at a fixed CO_2_ pressure (0.5 MPa), the yields increased from 6.2 to 97% with an increased temperature from 25 to 50 °C, but remained almost invariable with the temperature further increased to 80 °C (Figure 6). These results fully suggest that the activation of CO_2_ was not the rate-limit step, but the activation of carbon–carbon triple bonds by the Ag (I) ions. Additionally, the removal of **1** by filtration after 18 h shut down the reaction, and no additional conversions were observed for another 18 h under the same reaction conditions. This observation suggested that **1** features a typical heterogeneous catalyst nature in the catalytic system and is stable enough to ensure that no leaching of metal ions occurs during the catalysis process. The recyclability studies of catalyst **1** were also performed by simple filtration, washed with dichloromethane and dried, and reused for successive rounds with fresh Ph_3_P under the same conditions. The yields of cyclic carbonates were not significantly affected in the five catalytic cycles (Figure S9), ranging from 97% to 90%, although the loss of small amounts of the catalyst is unavoidable. IR spectroscopy of the catalyst after recycle reactions could match with the fresh catalyst (Figure S10), indicating the structural integrity and chemical stability of **1**.

Subsequently, several propargyl alcohols with various substituted R^1^ groups were performed for the carboxylative cyclization with CO_2_ under the same reaction conditions, and no significant change in the conversion was observed which all gave high yields, except for the CMe_3_ group, which showed a large decline in the reaction yield (78%, Entry 5). Moreover, changing a larger aromatic substrate in the substituted R^2^ group of propargyl alcohols could also cause the yield and TON value to obvious decreases (Entry 8). These results indicate that the electron-drawing or the electron-donating groups on the phenyl of propargyl alcohol have less effect on the reaction yields, and the main factor affecting the yield is the size of substrates.

**Figure 7:**
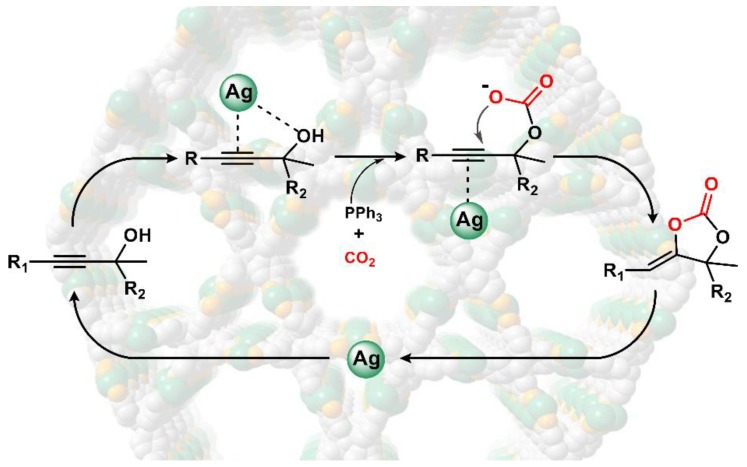
Plausible mechanism for the carboxylative cyclization from propargylic alcohols to cyclic carbonates with CO_2_ catalyzed by **1**.

It is postulated that the strong interaction between the substrate propargyl alcohol and our catalyst existed as a significant factor to enhance the high conversion of the catalytic reaction. Combining the analysis of dye absorption studies, we envision that the propargyl alcohol molecules were adsorbed and activated within the channels of our MOFs. Thus, the size of the channels is also an important factor that controls the efficiency of the conversion by influencing the transport of the substrates and products through the channels, which shows the size-selectivity in the catalytic reaction. Additionally, the unique decentralized silver (I) chains, which endow the specific alkynophilicity to activate C≡C bonds, and thus greatly accelerate the efficient conversion of CO_2_ to α-alkylidene cyclic carbonates in a heterogeneous manner. From a mechanistic point of view, the Ph_3_P plays a key role in the promotion of CO_2_ fixation and activation in the first step of the catboxylative cycloaddition, which the alcoholic hydroxyl was activated by the catalyst **1**, and CO_2_ was activated by Ph_3_P to form the carbonate intermediate (Figure 7). The O atom in hydroxyl group is more favorable to the electrophilic attack of the C atom in CO_2_. Subsequently, a nucleophilic attack to the silver propargylic carbonate intermediate that generated the Ag∙∙∙C≡C bonds by π-activation occurred for the next intramolecular ring-closing [35]. The corresponding carboxylative cyclization product is thus yielded with the process of proto-demetallation and the regeneration of Ph_3_P [36].

## 4. Conclusions

In conclusion, a porous Ag(I)-based polymer has been synthesized by incorporating the tetraphenylethylene moiety as the backbone, and proven to be an efficient heterogeneous catalyst for the carboxylative cyclization of CO_2_ to α-methylene cyclic carbonates in excellent yields. The large pores of **1** benefit for the adsorption of dye molecules and also allow the interaction and a fast transportation of the substrate molecule within their microenvironments. The introduced Ag (I) chains in the frameworks play an important role in the activation of subtract molecules for the internal alkynes by π-activation. This approach provided a highly promising method for the development of more practical industrial synthesis. Further work will focus on designing more efficient catalysts to optimize such reactions by improving the conversion and shortening the whole reaction times.

## Figures and Tables

**Table 1:** Crystal data and structure refinements.

Compound	1
Empirical formula	C_90_H_54_Ag_12_O_27_
Formula weight	2861.77
*T*/K	200(2)
Crystal system	Monoclinic
Space group	C 2/c
*a*/Å	31.9216(11)
*b*/Å	56.6381(18)
*c*/Å	11.0729(4)
*α*/^o^	90
*β*/^o^	97.905(2)
*γ*/^o^	90
*V*/Å^3^	19829.3(12)
*Z*	4
*D*_calc_/Mg m^–3^	0.959
*µ*/mm^–1^	1.191
*F*(000)	5496
*R*_int_	0.1206
Data/parameters	17,459/672
GOF	1.052
*R* [*I >* 2*σ*(*I*)] ^a^	*R*_1_ = 0.0747 *wR*_2_ = 0.2088
*R* indices (all data) ^b^	*R*_1_ = 0.1357 *wR*_2_ = 0.2376
Δ*ρ*_max,min_/eÅ^–3^	1.444/−0.783
CCDC number	1918824

^a^*R*_1_ = Σ||*F_o_*| − |*F_c_*||/Σ|*F_o_*|; ^b^ *wR*_2_ = Σ[*w*(*F_o_*^2^ − *F_c_*^2^)^2^]/Σ[*w*(*F_o_*^2^)^2^]^1/2^.

**Table 2:**
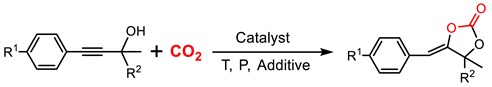
**1**-catalyzed cyclization of propargylic alcohols with CO_2_.

Entry	R^1^	R^2^	Yield	TON
1	H	H	97%	320
2	Me	H	94%	310
3	Et	H	93%	310
4	*n*-Pr	H	91%	300
5	*t*-Bu	H	78%	260
6	Cl	H	>99%	330
7	MeO	H	97%	320
8	H	Ph	28%	90

Reaction conditions: Propargylic alcohols (3 mmol), catalyst (0.3 mmol% loading, based on each Ag site), Ph_3_P (2.5 mmol%), and CH_3_CN (1 mL) under CO_2_ (0.5 MPa), 50 °C and 30 h. The yields were determined by ^1^H NMR analysis using durene as internal standard.

## Supplementary Materials

The following are available online at https://www.mdpi.com/2079-4991/9/11/1566/s1, Figure S1: the 3D structural framework of **1** along *a* axis, Figure S2: the 3D structural framework of **1** along *b* axis, Figure S3: the truncated 3D structure and schematic representation of **1** network, Figure S4: structure comparison of 1 in this work and the reported MOF PCN-222, Figure S5: PXRD patterns of **1** (red), its calculated pattern based on the single-crystal simulation (black) and the activated sample after desolvation (blue), Figure S6: TG curve of **1**, Figure S7: Gas adsorption isotherms of activated **1** of N_2_ at 77 K, Figure S8: The structure of RhB molecule used in the dye adsorption experiments, Figure S9: The recycling experiments of **1** at each round, Figure S10: IR spectra of **1** and the sample after reaction.
