# Supplementary material for: A Versatile Porous Silver-Coordinated Material for the Heterogeneous Catalysis of Chemical Conversion with Propargylic Alcohols and CO2
Source: Nanomaterials (Basel). 2019 Nov 5;9(11):1566. doi: 10.3390/nano9111566 (PMC6915374; doi:10.3390/nano9111566)
Supplement: Supplementary file 1 [file nanomaterials-09-01566-s001.pdf]

Supplementary Materials: A Versatile Porous Silver-Coordinated Material for the Heterogeneous Catalysis of Chemical Conversion with Propargylic Alcohols and CO_2

Lu Yang [1], Yong Dou [1], Zhen Zhou [1,2,*], Daopeng Zhang [1*] and Suna Wang [3]

[1] School of Chemistry and Chemical Engineering, Shandong University of Technology, Zibo, 255000, China; yanglu@sdut.edu.cn (L.Y.); douyongsdut@163.com (Y.D.)
[2] State Key Laboratory of Fine Chemicals, Dalian University of Technology, Dalian 116024, China
[3] Shandong Provincial Key Laboratory of Chemical Energy Storage and Novel Cell Technology, School of Chemistry and Chemical Engineering, Liaocheng University, Liaocheng 252059, China; wangsuna@lcu.edu.cn
* Correspondence: zhouzhen@sdut.edu.cn (Z.Z.); dpzhang73@126.com (D.Z.)

Contents

1. Supplementary Structural Figures.

2. Characterizations of Catalyst.

3. Catalysis Details.

1. Supplementary Structural Figures

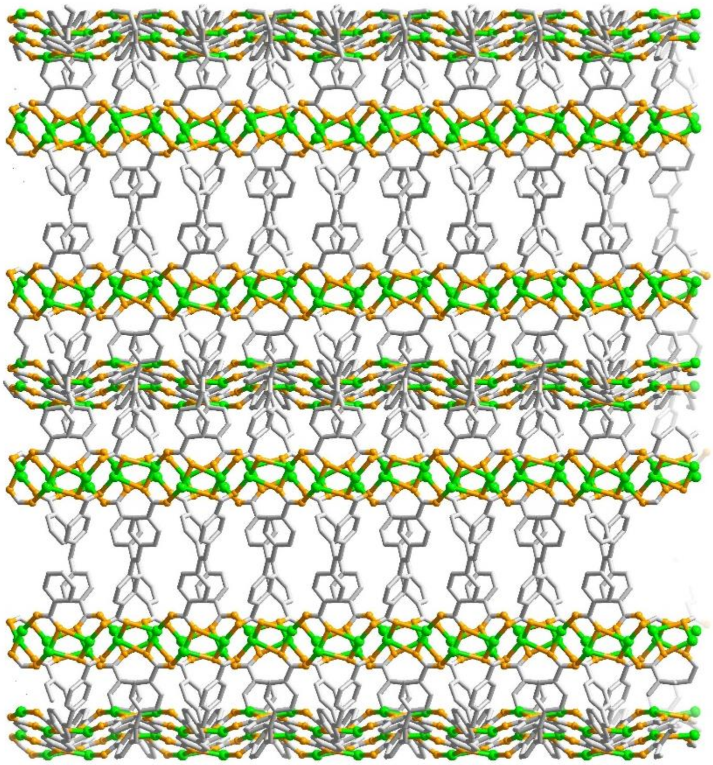

Figure S1. The 3D structural framework of **1** along *a* axis.

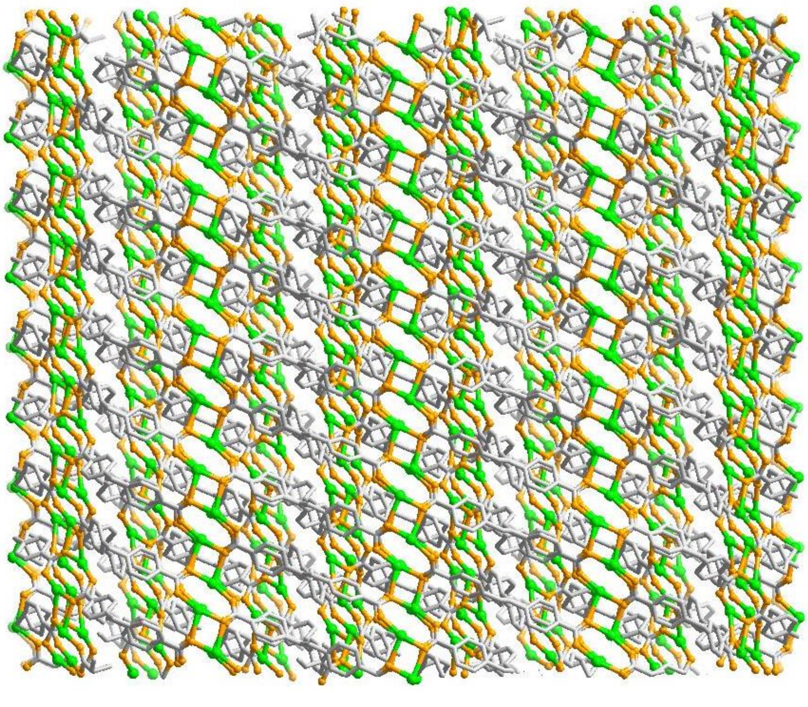

Figure S2. The 3D structural framework of **1** along *b* axis.

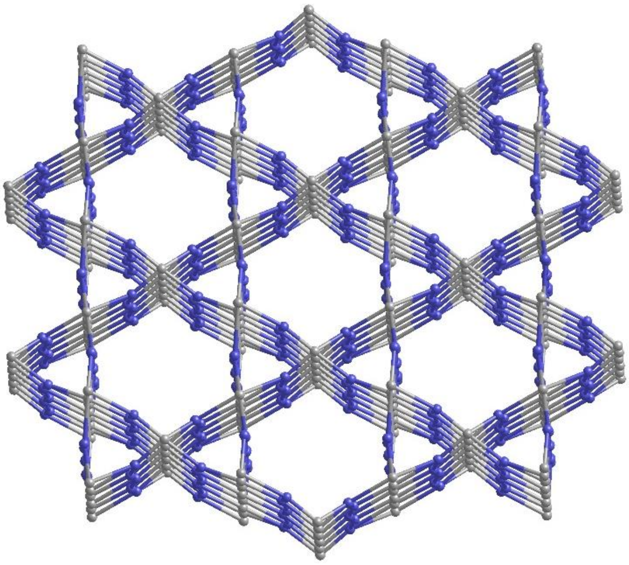

Figure S3. The truncated 3D structure and schematic representation of **1** network.

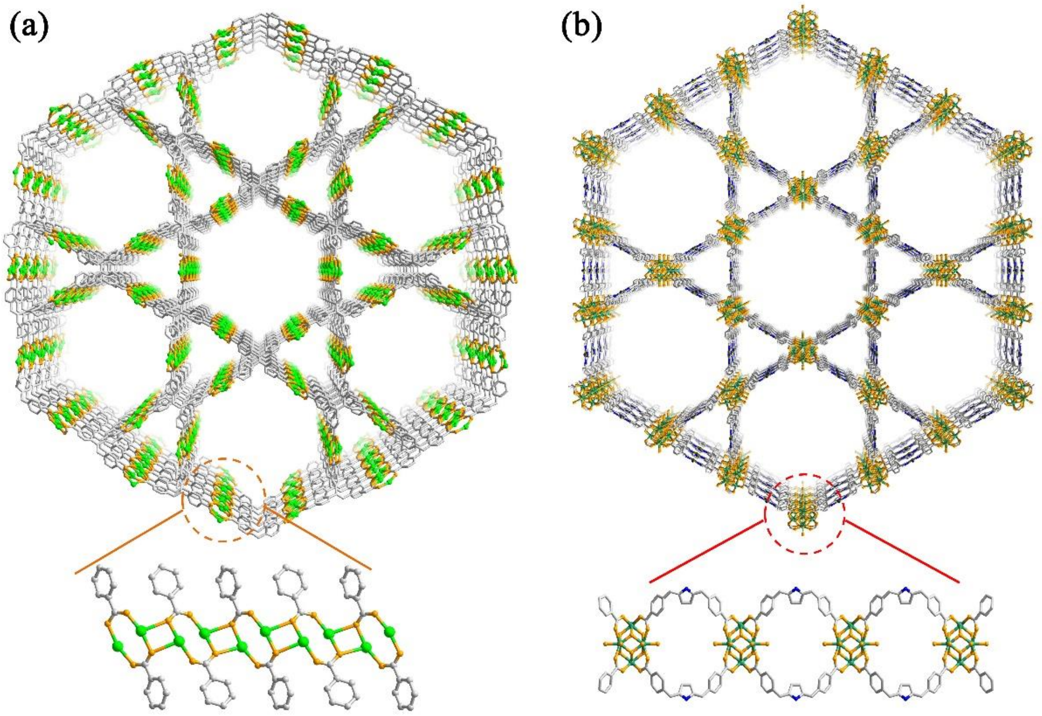

Figure S4. Structure comparison of **1** in this work and the reported MOF PCN-222.

2. Characterizations of Catalyst

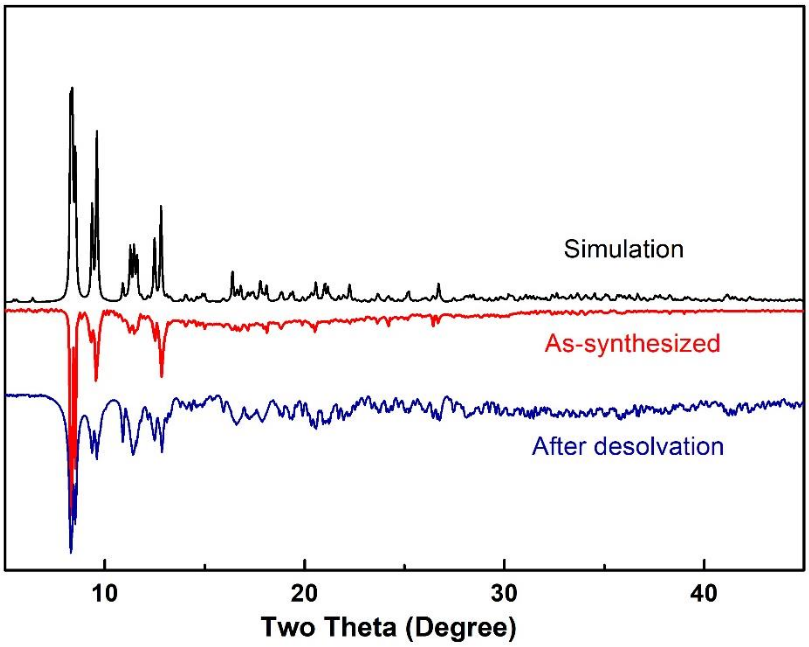

Figure S5. PXRD patterns of **1** (red), its calculated pattern based on the single-crystal simulation (black) and the activated sample after desolvation (blue).

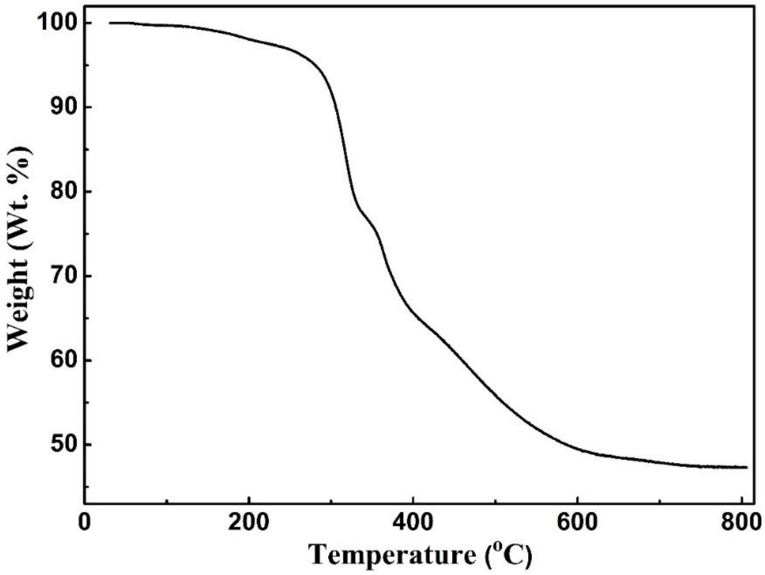

Figure S6. TG curve of **1**.

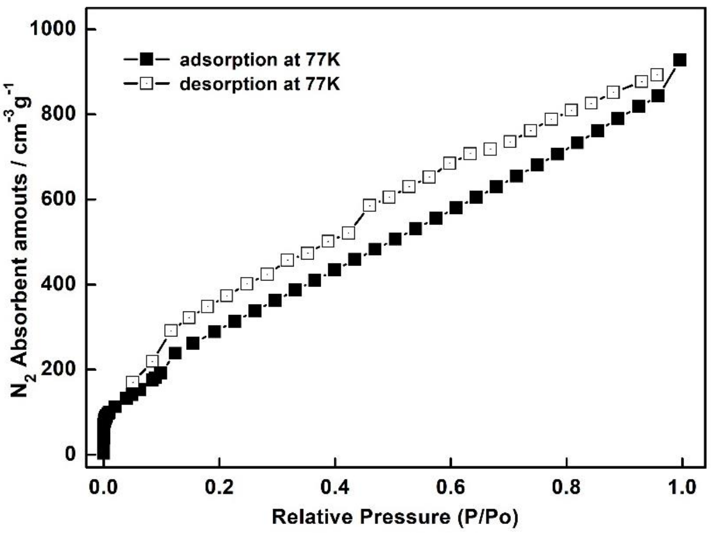

Figure S7. Gas adsorption isotherms of activated **1** of N_2 at 77 K. Filled shape, adsorption; open shape, desorption.

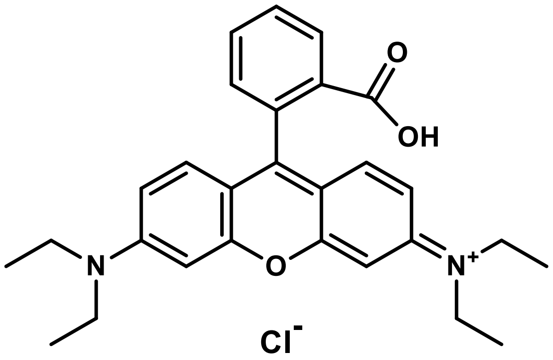

Figure S8. The structure of RhB molecule used in the dye adsorption experiments.

3. Catalysis Details

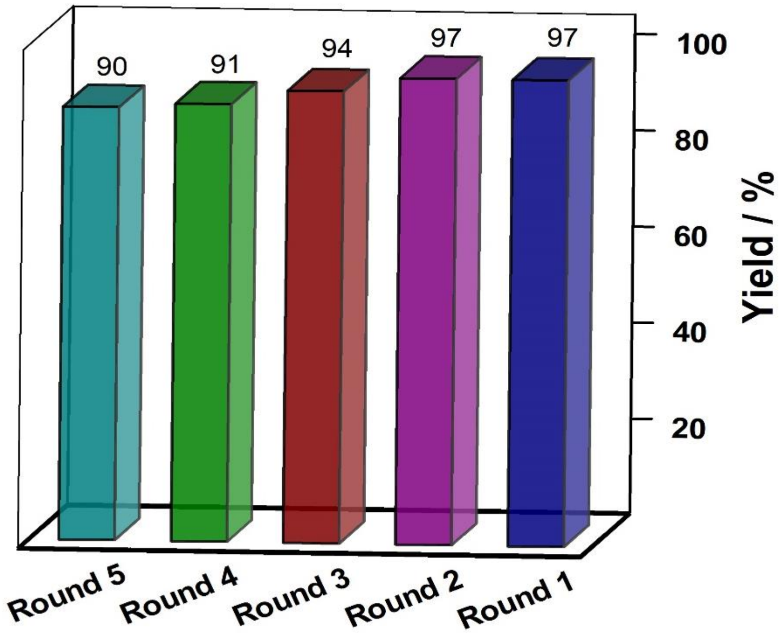

Figure S9. The recycling experiments of **1** at each round.

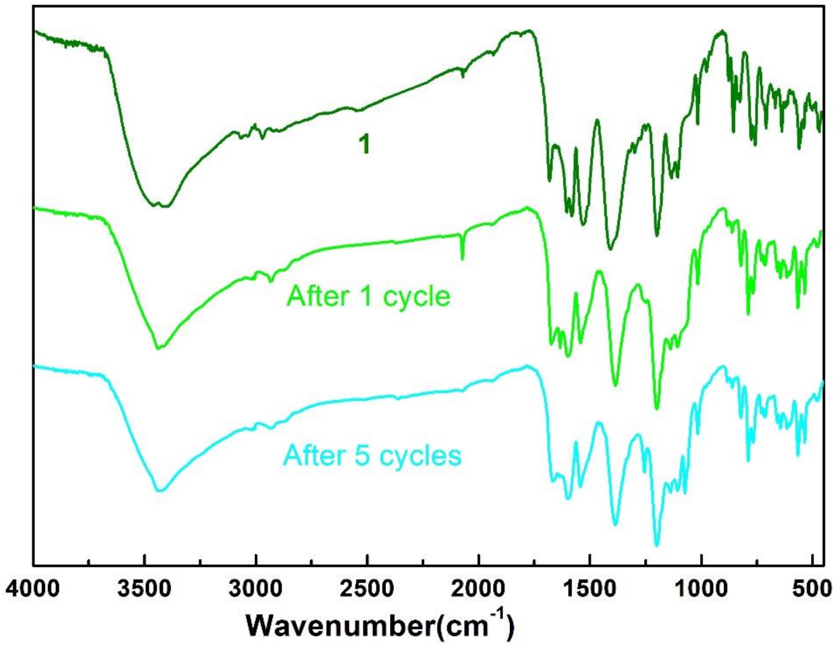

Figure S10. IR spectra of **1** and the sample after reaction.
